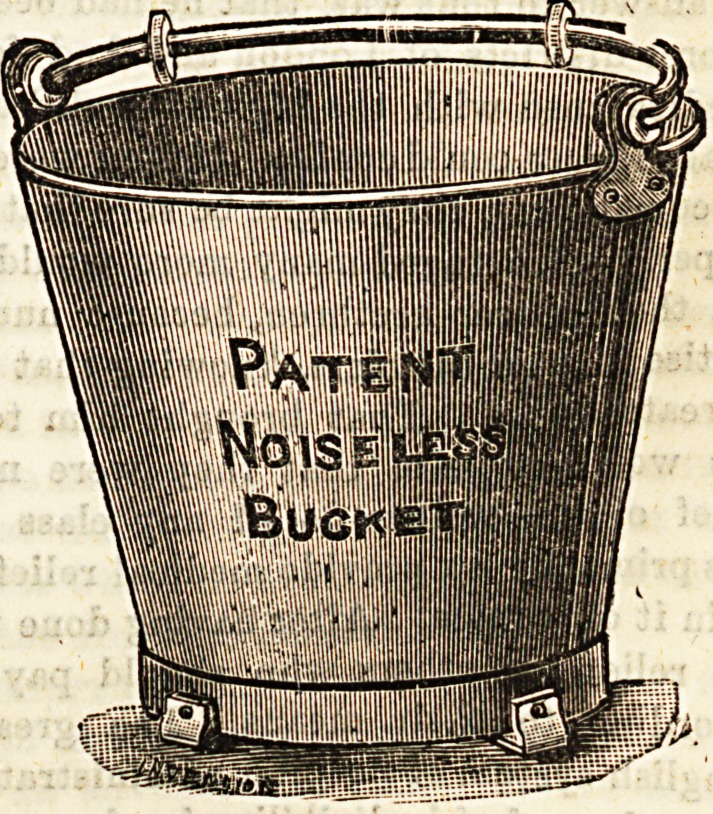# New Drugs, Appliances, and Things Medical

**Published:** 1892-05-07

**Authors:** 


### NEW DRUGS, APPLIANCES, AND THINGS MEDICAL.

[All preparations, appliances, novelties, etc., of which a notice is desired, should be sent for The Editor, to care of The Manager, 140, Strand, London, W.C.]

### A NOISELESS PAIL.

We have received from Messrs. Baxendale, Miller Street, Manchester, an article which will prove a real boon in households and institutions. This article is a noiseless pail. It has rubber feet, eyelets, and handle-guards, which are all durable, and so arranged that they cannot be put out of order—a very great consideration considering the class in whose hands the buckets will be placed. The invention is so admirable and of such obvious advantage, that it is quite surprising that we have stood the irritating noise of the usual zinc pail for so long. The price is only 1s. 6d., which is, we think, less than the public should expect to give for such an article, and we think it would be an added advantage if buckets of superior quality could be procured at a slightly higher price. No really economical housekeeper objects to pay a fair sum for objects in constant use. Quality is the cheapest investment in the end.

## Figures and Tables

**Figure f1:**